# Harmful chemicals emitted from electronic cigarettes and potential deleterious effects in the oral cavity

**DOI:** 10.18332/tid/116988

**Published:** 2020-05-08

**Authors:** Jeffrey Ebersole, Vera Samburova, Yeongkwon Son, David Cappelli, Christina Demopoulos, Antonina Capurro, Andres Pinto, Brian Chrzan, Karl Kingsley, Katherine Howard, Nathaniel Clark, Andrey Khlystov

**Affiliations:** 1 Department of Biomedical Sciences, School of Dental Medicine, University of Nevada Las Vegas, Las Vegas, United States; 2 Organic Analytical Laboratory, Division of Atmospheric Sciences, Desert Research Institute, Reno, United States; 3 Oral and Maxillofacial Medicine and Diagnostic Sciences, School of Dental Medicine, Case Western University, Cleveland, United States

**Keywords:** electronic nicotine delivery systems, e-cigarette, vaping, oral health, public health

## Abstract

Use of electronic nicotine delivery systems (ENDS), such as electronic cigarettes (e-cigs), is increasing across the US population and is particularly troubling due to their adoption by adolescents, teens, and young adults. The industry’s marketing approach for these instruments of addiction has been to promote them as a safer alternative to tobacco, a behavioral choice supporting smoking cessation, and as the ‘cool’ appearance of vaping with flavored products (e.g. tutti frutti, bubble gum, and buttered popcorn etc.). Thus, there is a clear need to better document the health outcomes of e-cig use in the oral cavity of the addicted chronic user. There appears to be an array of environmental toxins in the vapors, including reactive aldehydes and carbonyls resulting from the heating elements action on fluid components, as well as from the composition of chemical flavoring agents. The chemistry of these systems shows that the released vapors from the e-cigs frequently contain levels of environmental toxins that considerably exceed federal occupational exposure limits. Additionally, the toxicants in the vapors appear to be retained in the host fluids/tissues at levels often approximating 90% of the levels in the e-cig vapors. These water-soluble reactive toxins can challenge the oral cavity constituents, potentially contributing to alterations in the autochthonous microbiome and host cells critical for maintaining oral homeostasis. This review updates the existing chemistry/environmental aspects of e-cigs, as well as providing an overview of the somewhat limited data on potential oral health effects that could occur across the lifetime of daily e-cig users.

## INTRODUCTION

Tobacco use has decreased nationally over the last decades; however, electronic cigarette (e-cigs or electronic nicotine delivery system, ENDS) use is dramatically increasing in the US, especially among adolescents, teens and young adults where it has risen sharply since 2011^1,2^. E-cigs are handheld devices that produce an aerosolized mixture from a solution (i.e. e-liquid) typically containing nicotine, flavoring chemicals, vegetable glycerin (VG), and propylene glycol (PG), to be inhaled by the user^3^. The addictive nature of the e-cigs can be attributed to the nicotine levels in the device and the flavoring of the chemicals that target youth and young adults. Data from the 2011–2018 National Youth Tobacco Survey (NYTS) determined that current usage of e-cigs among high school students has risen from 1.5% in 2011 to 20.8% in 2018—a more than 13-fold increase. According to a 2013–2014 survey of adults and youth, flavored tobacco products predominantly attract young users (aged 12–24 years) and 81% of youth that currently use e-cigs cited appealing flavors as a primary reason for first using a tobacco product^4^. Research has shown that adolescents are more likely to experiment with substances such as cigarettes, and they are physically more vulnerable to addiction^5^. There are mixed views regarding the safety and efficacy of e-cigs, even among healthcare professionals. While some individuals view e-cigarettes as a public health concern, others recommend them as a safer alternative to conventional cigarettes for smokers who are unwilling/unable to quit. With the advent of new delivery systems and the addictive nature of nicotine, one can see how e-cigs can negatively impact the health of youth and young adults with potential for long-term impacts on oral health over a lifetime. However, the relative recent e-cig epidemic has not yet provided robust datasets to assess if systemic diseases result from the long-term usage of e-cigs. Regardless of the current debate on the use of e-cigs as an effective smoking cessation aid, the dramatic increase in e-cig usage among never smokers demands a clear need to better document the health outcomes of e-cigs in the oral cavity of the addicted chronic user.

There appears to be an array of environmental toxins in the vapors, including reactive aldehydes and carbonyls resulting from the heating elements action on fluid components, as well as from the composition of chemical flavoring agents. These reactive toxicants can challenge the oral cavity constituents, potentially contributing to alterations in the autochthonous microbiome and host cells critical for maintaining oral homeostasis. This review updates the existing chemistry/environmental aspects of e-cigs, as well as providing an overview of the somewhat limited data regarding potential oral health effects that could occur over the lifetime of daily e-cig users.

## DEVELOPMENTS

### Toxic/noxious end-products in e-cig vapors

As stated in the Surgeon General’s Report, *E-Cigarette Use Among Youth and Young Adults*, e-cigs contain varying levels of nicotine and other chemicals known to increase the risk of cancer^6^. According to Goniewicz et al.^7^, carcinogens, such as formaldehyde, acetaldehyde, and nitrosamines have been found in e-cig vapor. In addition, diacetyl is added to e-cigs, which destroys the airways in the lungs and can cause popcorn lung or bronchiolitis obliterans^8^. Findings suggest that e-cigs not only have systemic health concerns, but can also negatively affect the oral cavity. The chemical vapors produced by vaping can alter or damage the epithelial cells, leading to oral ulcerations or oral cancer^9^.

**Figure 1:**
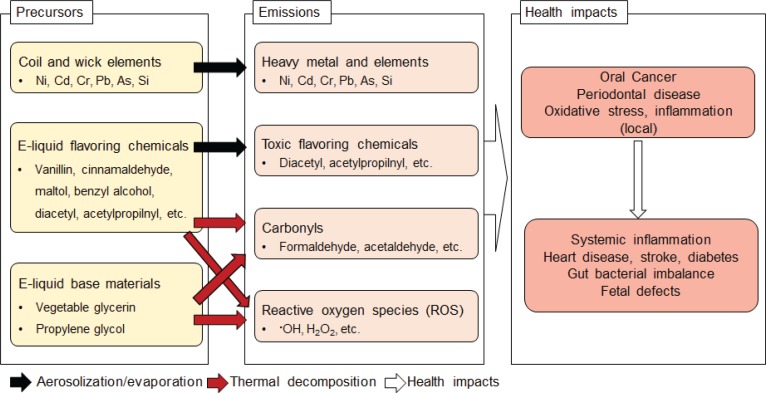
E-cig processes that contribute to potential toxicity for oral tissues

Toxic compounds, such as heavy metals, carbonyls, flavoring chemicals, and reactive oxygen species (ROS), have been detected in e-cig aerosols in concentrations that can adversely affect oral health (Figure 1). Some of these toxic compounds, such as diacetyl, can be found in some e-liquids, while others such as metals, carbonyls, and ROS, can form during e-cig use. During the vaping process, e-liquid is vaporized by a heating element operating at temperatures ranging between 100 and 300°C depending on the e-cig construction and power output. High temperatures facilitate transfer of heavy metals (e.g. nickel, cadmium, chromium, and lead) from the coil into the e-liquid^10^. E-liquid impurities and break-down of wick material may also lead to the presence of toxic elements such as arsenic and silica in e-liquids^11^. Aerosolization of e-liquid leads to emissions of these substances during vaping. A higher e-cig power output as well as aging of heating element wires could increase metal emissions^12^. Exposure to these metals is of concern as it can cause chronic periodontitis, oral cancer, inflammation, and neurodegeneration^11^.

E-cigs are also known to emit amounts of carbonyls (i.e. formaldehyde, acetaldehyde, acrolein, etc.) that depend on e-cig vaping conditions, such as vaping topography, power output, device construction, coil material, and e-liquid components. E-cig vaping topography, which refers to puff duration and volume, can affect processes occurring on the coil surface. A longer puff duration increases formation of toxic carbonyls under the same puff volume^13^. The same e-cig device produces significantly more carbonyls at a higher power setting due to the higher thermal decomposition rate of e-liquid components including PG, VG, and flavoring chemicals^14,15^. However, a better predictor of carbonyl emission strength is the ratio of applied power to the coil surface area: e-cigs with larger coils tend to produce less carbonyls per unit power output^16^. E-liquid composition also affects carbonyl emissions. At a fixed e-cig power output, PG-based e-liquids form more toxic carbonyls than VG-based e-liquids^17^. However, the main part of carbonyl compounds is due to the thermal decomposition of flavoring compounds^18,19^ or flavor-catalyzed decomposition of PG^20^. Carbonyl emissions also depend on the coil material, as metals in the e-cig heating element have been shown to accelerate thermal decomposition of e-liquid organic components triggering the formation of toxic carbonyl compounds^21^.

Formaldehyde levels in e-cig aerosols were reported to significantly exceed the occupational safety limits^10,15,18,22-24^. It has been debated that high carbonyl concentrations do not occur during normal e-cig usage and are generated only under so-called ‘dry-puff’ conditions that e-cig users avoid^25^. ‘Dry-puffs’ occur due to insufficient e-liquid supply to the wick leading to overheating of the liquid, resulting in a significant increase in toxic carbonyl emissions. However, a recent study noted high levels of toxic carbonyls in exhaled e-cig aerosols during normal e-cig usage^26^. Formation of ROS in e-cigs was also reported to depend on the kind of flavoring chemicals present in e-liquids^27,28^. Similar to carbonyl emissions, higher power outputs and longer puffs generate more hydroxyl radicals, the most destructive ROS species that can damage DNA, proteins, and lipids^28^. A VG-based e-liquid was reported to produce more hydroxyl radicals than a PG-based one^29^.

Toxic flavoring chemicals that cause ‘popcorn lung’ disease, diacetyl and acetylpropionyl, were detected in some milk-, butter-, fruit-, candy-, and cocktail-flavored e-liquids^30^. Sweet, chocolate, and cinnamon flavoring chemicals in e-liquids showed cytotoxic effects, oxidative stress, and inflammation responses in several *in vitro* studies^31-33^. *In vitro* toxicological e-cig studies are complicated due to the highly volatile and concentrated nature of e-cig aerosols and the difficulty to adequately capture the wide range of e-cig use patterns. Previous e-cig toxicological studies used smoking machines designed for conventional cigarettes and fixed vaping condition^33-35^. The exposure protocols in those studies were shown to be suboptimal because they alter e-cig aerosols and are unable to reproduce the wide range of e-cig vaping conditions. To overcome these limitations, an e-cig vaping machine was designed and used to generate e-cig aerosols at various vaping topography parameters to standardize the exposure protocol and achieve reproducible exposure results^36^. In another *in vitro* study, epithelial cells were exposed to undiluted fresh e-vapor using a 3D culture system^28^. This direct exposure system could maintain physical and chemical integrity of e-cig aerosol, mimicking the real-world exposure condition. Even though advances in e-cig toxicological study protocols have been made in recent years, local dosimetry of e-cig emissions needs to be studied further to identify local impact such as those on oral health.

In addition to *in vitro* studies, *in vivo* e-cig exposure studies are desired. A recent pilot study reported carbonyl retentions in e-cig users during e-cig vaping^26^. In 14 out of 19 cases, carbonyl levels in exhaled e-cig aerosols were 2–125 times higher than in pre-exposed breath. A significant fraction, 99.7±0.9% and 91.6±10% of carcinogenic formaldehydes and acetaldehydes, respectively, was retained by the users. High water solubility and reactivity of formaldehydes and acetaldehydes were shown to facilitate oronasal deposition of inhaled toxic carbonyls^37^. The high oronasal retention of formaldehydes and acetaldehydes could worsen oral health. There have been no reports yet on how inhaled e-cig formaldehydes and other toxic aldehydes are associated with oral diseases.

### E-cig use and oral health/disease

Studies of e-cig use continue to dissect out clinical impacts that include knowledge from animal models and cell biology studies to formulate an estimate of the magnitude of deleterious health consequences of e-cig use. While oral cancer outcomes of conventional cigarettes are well known, the role of e-cig use in this process has not been fully elucidated. With the increasing use of e-cigs, particularly in younger people, the long-term impact of this addictive behavior must be part of the repertoire of knowledge and actions of dental and oral health providers within the overall healthcare team^38^. Aldehyde adducts in tobacco smoke are clearly major factors in DNA damage and decreased repair, while acrolein also reduces DNA and protein repair processes^39^. E-cig aerosols induce DNA damage and decrease cellular antioxidant defences independent of nicotine on oral and lung epithelial cells^40^. Canistro et al.^41^ demonstrated the co-mutagenic and cancer-initiating effects of e-cig vapor in a rat lung model. Nicotine from e-cigs negatively impacted cell viability and proliferation of both cancerous and non-cancerous cells^42^. The role of e-cig derived nicotine on cellular functions including profibrotic response and other functional aspects is not known^43^. Thus, the knowledge base in this area still lacks robust data on the effect of vaping on the gingiva, and an evidence-base needs to be established^44^.

#### Cell biology

Although much remains to be discovered regarding the effects of various additives and by-products of e-cig vapors, a growing body of evidence has demonstrated cytotoxicity in the most likely affected cell types^45,46^. Acrolein, the simplest unsaturated aldehyde, has been demonstrated to be highly reactive, functions to cross-link DNA, and may be sufficient in e-cig vapors at concentrations that inhibit cytochrome P450 enzymes and induce apoptosis in a variety of lung and bronchial cells^47,48^. In addition, more recent evidence has suggested that reactive aldehydes, including acrolein, may induce ion channel dysfunction by reducing chlorine transport in airway epithelia^49,50^. Other cell types that may be affected by aldehydes, including formaldehyde and acrolein, include long basal epithelial cells, which acquired DNA strand breaks and other chromosomal damage at sub-cytotoxic concentrations, supporting observations of *in vitro* models^51-53^.

Further research supports these observations that reactive carbonyl species, such as the α and β-unsaturated aldehydes induce oxidative stress and increased protein carbonylation, which contributes to cardiovascular, pulmonary and oral cavity diseases and dysfunction^54,55^. Also, bronchial epithelial cells exhibit impaired ciliary function and cellular function in response to these e-cig components, which may be more pronounced when present in combination with nicotine^56,57^. However, the adverse cellular responses to e-cig aerosols are not limited to normal, healthy tissues but may also function to promote proliferation and transition to cancer in some tissue types^58-60^. Recent comparisons of e-cig users demonstrate elevated levels of carcinogens compared with controls, as well as the potentially higher risk of transformation of premalignant lesions and development of oral and esophageal cancers^61,62^. For example, e-cig consumers exhibit changes to oral mucosal lesions that may be comparable to those of smokers, and the effects of oxidative damage and other deleterious effects may exhibit more profound effects in premalignant and malignant lesions than in normal, non-cancerous tissues^40,63^.

More specifically related to reported effects on the oral cavity, e-liquid constituents increased cytotoxicity and apoptosis in human gingival fibroblasts, not related to nicotine content^64^. These types of e-liquids with or without nicotine also demonstrated cytotoxic and genotoxic effects on human oropharyngeal mucosa^65^ and showed varied effects on epithelial cell proliferation and viability that extended beyond the constituents of nicotine and propylene glycol/vegetable glycerin in the e-liquid fluids^66^. E-cig aerosols, likewise, reduced the viability and increased apoptosis and necrosis of epithelial cells unrelated to nicotine content^67^, as well as causing increased oxidative/carbonyl stress, inflammatory cytokines, DNA damage, and reduced HDAC2 responses in fibroblasts and gingival epithelium^9^. E-cig aerosols significantly decreased glutathione levels in oral keratinocytes leading to increased cytotoxicity^68^, and induced ROS, DNA damage and toxicity for vascular endothelial cells^69^, apparently reflecting changes in oxidative stress by components in these aerosols.

Beyond the potential direct effects on the critical epithelial barrier at mucosal surfaces, including the oral cavity, expanding literature has identified negative outcomes for e-cig vapor components on cells of the immune system. Cinnamaldehyde-containing e-cig liquids are broadly immunosuppressive for multiple immune cell types^50^. E-cig aerosol condensate was toxic to alveolar macrophages and increased ROS and inflammatory cytokines and chemokines, thus contributing to the inflammatory milieu in the lungs^70^. E-cig aerosols also increase pro-inflammatory cytokine production in models of human airways and decrease cell viability apparently unrelated to apoptotic processes^71^.

Thus, a combination of these deleterious outcomes driven by e-liquids and e-cig aerosols would be expected to demonstrate adverse effects on the health of the oral cavity. This concept continues to be supported by expanding literature in this field.

#### Periodontal disease

##### Microbiome

**Figure 2:**
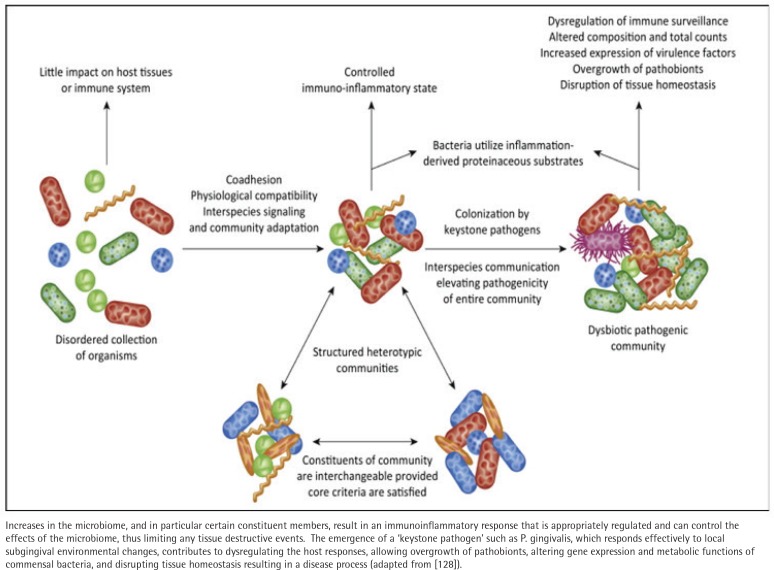
Schematic of the current paradigm in the microbiome of periodontitis, with a normal homeostatic microbiome comprising a large array of species of bacteria, which is symbiotic with host tissues and host responses

In humans, the interaction between animal and bacterial cells is especially important at mucosal surfaces. Methodologic advances have enabled rapid progress in characterizing the taxonomic composition, metabolic capacity, and immunomodulatory activity of the human microbiome, enabling insights into its role in health and disease. Yanushevich et al.^72^ suggested a consideration of ‘pathological colonization level’ as a concept that could be explored to better assess the complex etiologic factors in periodontitis. Defining the characteristics of the oral microbial ecology in periodontitis has also required the incorporation of various confounders, including age^73^, diabetes^74^, rheumatoid arthritis^75^, inflammatory bowel disease^76^ and sex^77^ as potentially modifying the oral microbiome in health and disease. However, no extrinsic modifier appears to have a greater effect on periodontal disease prevalence and severity than tobacco smoking^78^. While underlying critical triggers are unknown, current concepts of the transition of the ecology from health to disease now emphasize an altered balance of the oral microbiome, resulting in a ‘dysbiosis’^79^ (Figure 2). It still remains ill-defined how this dysbiosis is created and whether this is primarily being driven by an emergence of pathogenic bacteria in the subgingival ecology and/or stimulation of a dysregulated host innate and inflammatory response that is modulated by genetic and epigenetic predisposition, as well as patient-modifiable factors including smoking, diet, diabetes, stress etc.^79^ In this regard, little current information is available on the impact of e-cigs in altering the oral microbiome to increase disease risk. Thus, the stage has been set with years of investigation and dramatic improvements in technologies to better understand the array and organization of a homeostatic microbiome so as to delineate critical changes that might occur that are driven by the use of e-cigs.

##### Mucosal responses

Mucosal tissues are colonized by an extremely dense and diverse microbiota of commensal bacteria, and are often the first sites of interaction with pathogenic microorganisms^80,81^. The first line of defence is sentinel cells consisting of macrophages, dendritic cells (DCs), and granulocytes patrolling for evidence of microbial challenge or infection. These cells effectively engage microbes using a repertoire of pattern recognition receptors (PRRs)^82^, which recognize distinct classes of microorganism-associated molecular patterns (MAMPs), including a range of bacterial, viral, and fungal pathogen ligands^83^.

**Figure 3:**
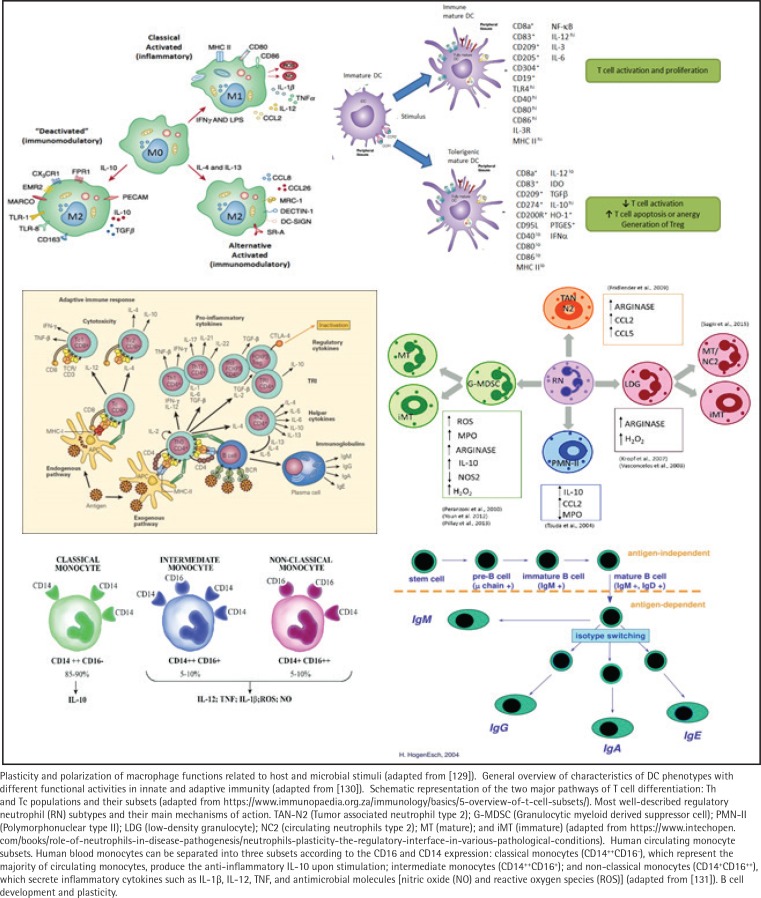
Immune system plasticity

Recent evidence has emphasized the plasticity of numerous immune cell types related to protection from infection, regulation of phenotypes and functions of inflammatory and immune responses, and development of tumors (Figure 3). These variations are regulated by the types of microorganisms providing the stimulus and the local host microenvironment^84^. The resulting signaling pathways activated through these receptors and processes lead to different immune cell response patterns, including macrophages, dendritic cell subsets, neutrophil subpopulations, seven different CD4+ T cell subsets, and B-lymphocyte heterogeneity.

A summary of existing reports demonstrates that this array of phenotypes of immune cells are present in the periodontium, respond to the environment at diseased sites, and likely contribute crucial functions to maintaining or re-establishing homeostasis of the oral tissues. The regulatory processes needed for homeostasis and dysregulation of these cell types with disease is of particular importance at mucosal surfaces as they are in constant association with external antigenic stimuli including pathogenic biofilms. Thus, the profile of cellular plasticity variations related to health or disease remains to be determined; however, studies exploring the role of plasticity in the pathogenesis of chronic inflammatory diseases and the potential altered cell repertoire elicited by stressors, such as those contained in e-cig products, are clearly needed.

### E-cig (ENDS) effects

The literature remains less than robust regarding long-term e-cig usage and effects on oral health. However, clear data on conventional cigarette use, and also on waterpipe tobacco use, which is becoming increasingly popular with younger people, associated with periodontitis, premalignant oral lesions, and oral and esophageal cancers, are lacking^62^. Clinical oral data with e-cig use are not clear. Periodontal inflammation was found to be decreased in cigarette and e-cig users compared to non-smokers, and self-perceived oral symptoms were worse in cigarette smokers than in e-cig users^85^. Similarly, following full mouth ultrasonic scaling, gingival inflammation was found to be elevated in cigarette smokers compared to e-cig users and non-smokers^86^. Moreover, other reports suggest that clinical measures of periodontal disease are no different in e-cig versus never smokers and greater in tobacco smokers. Similar results were found with salivary levels of some inflammatory mediators^87^. E-cigs vaporize a mixture of PG/VG, nicotine and flavoring agents with some marketing focus as an aid in smoking cessation with limited data of self-report and oral exams suggesting some improvement over tobacco smoking^88^.

However, other investigations have indicated that pathophysiological changes occur with e-cig aerosols including oxidative stress, DNA damage, altered innate host responses, inflammation, cellular senescence, profibrinogenic and dysregulated repair that could contribute to oral disease including periodontitis^85^. Formaldehyde toxicity also was reported to disrupt the functions of the periodontium, including alveolar bone, and altered cell growth and remodeling factors in rats^89^. Finally, elevated levels of proinflammatory cytokines are detected in crevicular fluid of dental implants in cigarette and e-cig users^90^.

#### Orthodontic complications

Orthodontic tooth movement has been well characterized related to the induced inflammatory response that focuses activation of osteoblasts or osteoclasts resulting in differential bone formation and resorption, enabling the tooth to move. Various more recent approaches have attempted to utilize knowledge of the biology of these processes to enhance the capability for more rapid and reproducible movement. However, within the context of the mechanical aspects of this treatment, multiple ‘confounders’ can affect the quality of the outcome. Within the oral microbiome, there are differences that occur in the quantity and quality of the microbial members related to the bonding of brackets to the teeth, or even with the more recent adoption of the clear aligner treatment technology. These microbiome changes have been associated with both white spot and risk of carious lesions, as well as an accretion of bacteria that can trigger untoward inflammatory responses. Thus, another component is the regulation or dysregulation of inflammatory responses that are necessary for tooth movement, but may pose a risk for eliciting periodontitis. Finally, there are clear data regarding an increased incidence of external apical root resorption (EARR) that likely has both genetic and environmental contributors^91^.

Although there are no studies that have evaluated the complex mixture of e-vapors and orthodontic tooth movement, available studies related to this topic are those that have examined the effect of nicotine alone on bone resorption related to orthodontic tooth movement. Several of these studies have demonstrated that nicotine administration increases the rate of orthodontic tooth movement in a dose-dependent manner^92-95^. Accelerated tooth movement is often considered desirable in orthodontic treatment; however, the application of orthodontic force with exposure to nicotine causes a significant increase in periodontal bone loss compared to orthodontic force alone^96^.

##### Microbiome

Various oral bacterial, including uncommon species, and fungal species are increased significantly after application of fixed/removable orthodontic appliances^97-100^. Greater microbial diversity was noted in patients with orthodontic appliances by 10–12 months, accompanying some differences in species distribution between the controls and orthodontic treatment^101^, although some decreases in the microbiome diversity were observed in clear aligners^102^. Finally, following fixed orthodontic bracket removal, decreases in several oral pathogens were observed and were related to gingival bleeding and plaque levels^103^. A broad array of bacterial taxa was identified with white spot lesions (WSL), as well as with gingivitis in children with fixed appliances. Adjustment for gingivitis did not alter the taxa associated with WSL, and certain taxa were more strongly related to gingivitis^104^. *C. albicans* was also increased in saliva and plaque samples in patients with white spot lesions formed during multi-bracket orthodontic appliance treatment^105^. Furthermore, recent systematic reviews supported increases in *S. mutans* and *Lactobacillus* spp., as well as potentially pathogenic Gram-negative oral species following orthodontic appliances^106^. Additionally, selected oral periodontal pathogens were elevated after appliance placement with levels decreasing a few months post-removal of the brackets^107^.

While virtually no data are available regarding e-cig use and microbiomes with orthodontic therapy, as the increased usage of e-cigs overlaps with the patient age range seeking orthodontic treatment, based on existing literature of e-cigarette effects on the oral microbiome, one must ask clinical questions regarding the potential longer-term deleterious consequences of vaping on successful orthodontic therapy.

##### Inflammation

Regarding the details of gingival inflammation related to orthodontic tooth movement that could be affected by the use of e-cigs, increased plaque levels were noted after bracket bonding and major increases in gingivitis measures were routinely observed^100^. Within three months of bracket placement, bleeding on probing, plaque index and gingival index were significantly increased, with multiple putative periodontal pathogens elevated and related to the increased inflammation^97^. In contrast, rather minimal increases in plaque and gingival bleeding were noted in patient’s after clear-aligner placement^102^. Several cell types responsible for the maintenance of alveolar bone and orthodontic tooth movement are adversely affected by exposure to nicotine. *In vitro* studies using PDL fibroblasts have demonstrated increased expression of COX-2, PGE2, IL-6 and RANKL with a simultaneous decrease in osteoprotegerin (OPG) expression during nicotine exposure.

Beyond these rather limited observations, the breadth of the population accessing orthodontic tooth movement and the increasing number of individuals addicted to e-cigs might be anticipated to result in an increased prevalence of adverse outcomes of the orthodontic treatment in this subset of the population.

##### Bone biology

The balance between bone formation on the tension side and resorption on the compression side of a moving tooth is critical to achieving net movement when orthodontic forces are applied. Nicotine disrupts this balance by suppressing osteoblast proliferation and inducing osteoblast apoptosis resulting in an overall decrease in osteoblastic activity on the tension side of teeth subjected to orthodontic forces^93,108-110^. This imbalance results in increased alveolar bone resorption around moving teeth and acceleration of tooth movement^95^. The increases in osteoclastogenic differentiation of osteoclast precursors and resorption activity of mature osteoclasts by nicotine appear to be mediated by changes in RANKL-RANK signaling and the expression of TNFα and PGE_2_^111^.

Orthodontically-induced inflammatory root resorption (OIRR) is also an undesirable effect observed with accelerated orthodontic tooth movement due to the administration of nicotine^94^. Using an *in vivo* rat model, significantly more root resorption was observed with increased odontoclastogenesis and expression of RANKL with nicotine exposure^112^. Considering that RANK/RANKL signaling regulates both bone resorption by osteoclasts and root resorption by odontoclasts, it is not surprising that both are affected by nicotine exposure. Finally, a recent report mentioned that e-liquids can lead to osteotoxicity, primarily via effects on osteoblasts. The effects were flavor-dependent and independent of nicotine^113^.

The inflammatory changes and bone altering biomolecules associated with nicotine in e-cig vapor would result in increases in OIRR and periodontal bone loss, compromising the oral health of patients and stability of the final orthodontic result. The associated risks of nicotine and e-cigs should be discussed with prospective orthodontic patients and treatment should be delayed until the patient has ceased all nicotine consumption.

#### Other Oral Conditions

##### Oral lesions

The occurrence of oral mucosal lesions in e-cig users was compared to previous smokers in a small sample recruited over two years^63^. The overall prevalence of oral mucosal lesions was 61% (n=55). Forty-five per cent of these were fungal infections, with 16 cases reported in the e-cig group. There was no significant difference between the two groups in terms of the frequency of lesions. Of interest, the presence of hyperplastic candidiasis on the commissure area in the e-cig users in this study was hypothesized to be associated with the process of heat vaporization and non-nicotine elements released into the perioral area. Other commonly reported lesions in the study for e-cigs were nicotinic stomatitis and hairy tongue. *In vitro* evidence describes increased pathogenicity of *Candida albicans* when exposed to commercial e-vapor^114^. This effect was mediated by an increased expression of chitin and secretory aspartate proteases (SAP2, 3 and 9) and phenotypic changes such as increased hyphal length. Direct comparison with non-exposed C. albicans cultures not exposed to the vapor highlighted significant interactions and enhanced adhesion of the fungus to gingival cells. This report enforces the still preliminary, yet substantive evidence, about a higher risk of fungal infection in e-cig users versus traditional smokers.

Aldehydes found in components of e-cigs are also linked to a higher risk of autoimmune reactions^115^. A murine model demonstrated an increase in autoimmune markers in mice exposed to perchloroethylene in the water at 12, 18 and 24 weeks, including levels of serum ANA (anti-nuclear antibodies), dsDNA (double-stranded DNA) and Scl-70 (scleroderma) antibodies. This increase was time-dependent and accompanied by a decrease in antioxidants mediated by lipid-derived aldehydes. The potential for autoimmune changes may influence the future occurrence of oral lesions with expanded long-term usage of e-cigs.

##### Mucosal pain

Perhaps one of the most intriguing areas in regard to adverse effects of e-cigs is mucosal irritation/pain. A population-based survey of high school students conducted in Korea explored the prevalence of gingival, tongue, and buccal mucosa pain in users of e-cigs versus controls^116^. Among 33309 responders, e-cig users were at a 54% higher risk of developing tongue and buccal mucosa pain. Additional evidence on the effect of nicotine and the possible effect of aldehydes (cinnamaldehyde) on pain receptors has been reported in controlled human studies^117^. Nicotine functions as an activator of the transient receptor potential subtype A1 (TRPA1) channel, associated with oral burning. This preliminary study reported subjects sensitized to the aldehyde reporting more burning complaints when exposed to nicotine. Both cinnamaldehyde and nicotine also alter the vasomotor activity in the oral and pharyngeal mucosa, and chronic exposure may modify pain reception in these areas^118^. To our knowledge, no publication has addressed this effect in e-cig users. With variability in nicotine content of e-cigs, the impact on oral pain perception may be higher than cigarette users. Another study reported no significant differences in gingival pain (as a secondary outcome) between e-cig users and controls. This study used a convenience sample and reported periodontal measures as the primary outcome^119^.

This physiological underpinning may support the phenotype of burning mouth syndrome, an oral pain disorder associated with several neuropathic diseases, and possibly hematinic or endocrine deficiencies. Some cases, particularly those only involving the tongue in the presence of marginal salivary flow, may be related to the effect of nicotine on specific pain receptors in smokers. Several studies identify cigarette smoking as an independent factor associated with this condition^120^.

##### Drug-induced hyposalivation

The primary efficacy of e-cigs on the reduction of craving was demonstrated in several recent systematic reviews^121^. As previously stated, many of these effects are mostly unknown and possibly related not only to nicotine but also to other substances mixed within e-cigs^122^. Dry mouth, irritation, and throat symptoms are among frequently reported adverse effects of chronic usage of e-cigs. However, these adverse effects decrease with prolonged usage of e-cigs^123^. Most studies based these conclusions on self-report of dry mouth. To date, no publication has addressed objectively whole or glandular specific salivary hypofunction in e-cig users. E-cigs may transiently increase mucosal blood flow in the oral cavity. The clinical significance of this finding remains to be confirmed^124^. It certainly is true that nicotine in cigarettes may cause swelling and inflammation of the palatal minor salivary glands. Because levels of nicotine in e-cigs vary depending on the brand, adverse effects on minor salivary glands may be underreported. Further, hyposalivation is a significant risk factor for the development of oral fungal infections, which may be the underlying etiology for the reported prevalence of these lesions in both cigarette smokers and e-cig users.

##### Type 2 Diabetes Mellitus (T2DM)

While T2DM is clearly a general disease affecting many tissues across the body, it is clear that oral complications of this disease dramatically impact the overall health and quality of life of affected individuals. Exposure to nicotine in cigarettes and e-cigs affects endothelial function, promotes inflammation and oxidative stress, and increases the risk of developing glucose intolerance and T2DM^125^. Also, the blood-brain barrier is affected, leading to cerebrovascular disease. Common medications for T2DM, metformin and rosiglitazone have shown *in vitro* upregulation of Nrf2, a related factor to the nuclear factor erythroid 2 and a potent antioxidant, anti-inflammatory that decreases endothelial damage^125^. The former medicine has been demonstrated in the same study to protect brain-blood barrier integrity. Albeit preliminary, this and other reports highlight potential *in vivo* implications for therapeutics targeting T2DM patients who also smoke or use e-cigs.

Another aldehyde, alpha-oxoaldehyde (MG), plays a major role in structural protein and receptor changes involved in the generation of advanced glycation end products (AGES), the main cytotoxic components of the diabetic process^126^. Modified MG-IgG appears to be a potent oxidative and immune reactive molecule, potentially implicated in the cascade of glucose intolerance and overt T2DM. The fraction of this specific aldehyde in e-cigs is uncertain. However, there is ample evidence that T2DM patients are at a higher risk to develop cancer due to sustained hyperglycemia, while generation of AGEs via MG is implicated in the pathogenesis of renal, retinal and neuropathic complications in murine models of T2DM^127^. Further, generation of AGEs via MG may be responsible for the higher risk of cancer in these individuals^127^.

## Conclusion

The past decade has seen a significant rise across the US in the use of e-cigs, especially among younger people. Recent studies have shown the presence of numerous potential carcinogens in e-cigs including nitrosamines and reactive carbonyls. Adducts from the heated flavoring components have been shown to have deleterious effects on host cells. Additionally, not only are the e-cigs an effective means for delivering high doses of nicotine but also may adversely affect oral health.
